# The emerging role of CETP inhibition in the prevention of Alzheimer's disease^☆^

**DOI:** 10.1016/j.ajpc.2026.101442

**Published:** 2026-02-16

**Authors:** Michael H Davidson, Andrew Hsieh, Mathijs de Kleer, Michael S. Szarek, Philip Scheltens, Everard Vijverberg, Adam Johnson, Marc Ditmarsch, John J.P. Kastelein

**Affiliations:** a NewAmsterdam Pharma, Amsterdam, the Netherlands; b University of Colorado Anschutz Medical Campus and CPC Clinical Research, Aurora, CO, USA; c Mount Sinai Fuster Heart Hospital, Icahn School of Medicine at Mount Sinai, NY, NY, USA; d State University of New York Downstate School of Public Health, Brooklyn, NY, USA; e Alzheimer Center Amsterdam, Neurologie, Vrije Universiteit Amsterdam, Amsterdam University Medical Center, Amsterdam, the Netherlands; f EQT Life Sciences, Amsterdam, the Netherlands; g Amsterdam Neuroscience, Neurodegeneration, Amsterdam UMC location VUMC, Amsterdam, the Netherlands

**Keywords:** CETP inhibition, Alzheimer's disease, APOE4, cardiovascular disease, lipid metabolism, p-tau217

## Abstract

We recently showed that patients with atherosclerotic cardiovascular disease (ASCVD) carry a substantial but largely unrecognized burden of early Alzheimer's disease (AD) pathology. In the BROADWAY pivotal phase 3 lipid-lowering trial, nearly half of participants with high-risk ASCVD had plasma p-tau217 concentrations above thresholds associated with preclinical AD, yet none had undergone evaluation for cognitive impairment. In this population, apolipoprotein E ε4 (APOE4) carriers were disproportionately represented among those with the highest p-tau217 levels. These findings expose a critical gap between cardiovascular care and dementia prevention and raise the question whether interventions targeting shared pathophysiology could address both conditions simultaneously.

Cholesteryl ester transfer protein (CETP) inhibition has emerged as a candidate for this dual role. In BROADWAY, obicetrapib reduced p-tau217 progression across the study population, with effects most pronounced in APOE4 carriers. In fact, treatment differences favoring obicetrapib were observed across all measured AD biomarkers in high-risk subgroups, including neurofilament light chain, glial fibrillary acidic protein, and the amyloid-beta (Aβ) 42:40 ratio. Unlike approaches that target downstream pathology, such as amyloid plaques already deposited in the brain or the inflammatory consequences of established disease, CETP inhibition may address the upstream processes involved in initiating the pathological cascade: lipid dysregulation, cholesterol ester accumulation in glial cells, impaired cholesterol efflux, lipid peroxidation, oxysterol formation, and deficient antioxidant transport.

This review examines the biological rationale linking APOE4 status to disordered lipid metabolism in both peripheral and central compartments, the genetic and epidemiological evidence supporting CETP as a therapeutic target, the mechanisms through which CETP inhibition might confer neuroprotection, and the clinical data suggesting obicetrapib as the first oral agent associated with favorable changes in AD biomarkers across both amyloid and tau axes in individuals at high genetic risk for the development of AD.

## 1 Introduction

The intersection of cardiovascular disease and Alzheimer's disease (AD) represents one of the most consequential yet underrecognized challenges in contemporary medicine [1]. Recent observations from the BROADWAY trial have exposed a burden of preclinical AD pathology among patients with atherosclerotic cardiovascular disease (ASCVD) who are otherwise receiving regular medical care. In a prespecified biomarker analysis of 1,535 participants with established ASCVD or heterozygous familial hypercholesterolemia (HeFH), nearly half demonstrated plasma phosphorylated tau-217 (p-tau217) concentrations exceeding 0.42 pg/mL, a threshold associated with high sensitivity and specificity for detecting emerging AD pathology, yet none were under neurological care or had received cognitive screening [2].

This hidden cognitive vulnerability was not uniformly distributed. Apolipoprotein E (APOE) ε4 carriers were disproportionately represented among those with the highest p-tau217 levels: one-third of patients with p-tau217 exceeding 0.62 pg/mL, a threshold with high sensitivity and specificity for established AD pathology, carried at least one E4 allele, compared with 18% of those below the 0.42 pg/mL threshold [2]. These findings align with prior work demonstrating that approximately 29% of ASCVD patients exhibit cognitive performance consistent with mild cognitive impairment (MCI) despite lacking formal diagnoses, and over half have p-tau217 levels exceeding age-matched population estimates. ASCVD populations face substantial, clinically silent AD risk (Table 1) [3].

**Table 1:** APOE genotype distribution by baseline p-tau217 status in BROADWAY.

APOE Genotype	>0.42 pg/mL (n=730)	≤0.42 pg/mL (n=859)	% with elevated p-tau217
E2/E2	4 (0.5%)	2 (0.2%)	66.7%
E2/E3	33 (4.5%)	69 (8.0%)	32.4%
E2/E4	12 (1.6%)	9 (1.0%)	57.1%
E3/E3	457 (62.6%)	624 (72.6%)	42.3%
E3/E4	202 (27.7%)	147 (17.1%)	57.9%
E4/E4	22 (3.0%)	8 (0.9%)	73.3%

Values are n (% of column total). APOE ε4 carriers (E3/E4 and E4/E4) comprised 30.7% of participants with elevated p-tau217 versus 18.1% of those with normal levels. E4 homozygotes showed the highest rate of elevated p-tau217 (73.3%).

In addition, the BROADWAY trial demonstrated that CETP inhibition with obicetrapib significantly modified AD biomarker trajectories over 12 months of treatment. Among APOE4 carriers, obicetrapib reduced p-tau217 progression by 5.74% relative to placebo (P=0.022), with effects intensifying in higher-risk subgroups: carriers aged 60 years or older showed 5.40% relative reductions (P=0.06), those aged 70 or older demonstrated 8.39% reductions (P=0.039), and homozygous E4/E4 carriers exhibited the most pronounced effects at 20.48% (P=0.010). These effects extended across the full spectrum of AD biomarkers in E4/E4 carriers: neurofilament light chain (NfL), a marker of axonal injury, showed a 17.31% difference favoring obicetrapib (P=0.020); glial fibrillary acidic protein (GFAP), reflecting astrocyte activation and neuroinflammation, showed a 15.24% difference (P=0.006); and the Aβ42:40 ratio demonstrated a 7.96% difference favoring obicetrapib (P=0.013). These data represent the first demonstration of an oral intervention associated with favorable changes in biomarkers across both amyloid and tau pathology in individuals at high genetic risk for AD [2].

It should be noted that the AD biomarker analysis in BROADWAY was a prespecified substudy; however, the trial was not powered for cognitive outcomes, follow-up was limited to 12 months, and cognitive assessments were not performed. While the biomarker effects are biologically coherent and statistically significant, they require confirmation in dedicated trials with longer duration and cognitive endpoints.

The mechanistic significance of these findings becomes evident when contrasted with recent failures of alternative approaches. In November 2025, Novo Nordisk announced that two large phase 3 trials, EVOKE and EVOKE-Plus, failed to demonstrate clinical benefit with oral semaglutide in over 3,800 patients with early symptomatic AD. Despite strong preclinical rationale for glucagon-like peptide-1 (GLP-1) receptor agonism in neurodegeneration, semaglutide produced no significant effect on plasma p-tau217 and was associated with increases in both plasma GFAP and NfL. The therapeutic hypothesis underlying GLP-1 agonism centered on reducing neuroinflammation through decreased neutrophil infiltration into the central nervous system (CNS), attenuated astrocyte activation, and improved peripheral metabolic function [4,5]. These results suggest that targeting inflammatory consequences without addressing upstream metabolic causes may prove insufficient, though important differences in trial design, patient populations, and disease stage between the semaglutide and obicetrapib studies preclude direct comparison. The GLP-1 trials enrolled patients with early symptomatic AD, whereas BROADWAY assessed biomarker effects in a cardiovascular population without clinical dementia, representing fundamentally different points along the disease continuum.

This distinction provides a fundamental principle in AD prevention. Neuroinflammation in APOE4 carriers does not occur spontaneously; it represents a downstream response to a primary metabolic dysfunction, the accumulation of lipid droplets within glial cells that disrupts their normal function and triggers reactive inflammatory cascades. Oxidative stress and neuroinflammation are intimately linked, each amplifying the other in a vicious cycle, with accumulating evidence pointing to oxidized cholesterol as a central driver of AD pathogenesis [6]. Suppressing this inflammatory response without correcting the underlying lipid dysregulation appears insufficient to modify disease course. Neuroinflammatory responses also serve protective functions, facilitating clearance of protein aggregates, maintaining blood-brain barrier (BBB) surveillance, and coordinating reparative processes [7]. The increases in NfL and GFAP observed with semaglutide suggest that peripheral metabolic modulation cannot adequately address the central lipid trafficking deficits that drive APOE4-associated pathology.

CETP inhibition represents a mechanistically distinct approach that may target upstream metabolic disturbances directly. Rather than suppressing inflammation as a primary mechanism, CETP inhibition addresses the lipid dysregulation that drives inflammatory activation in the first place, allowing neuroinflammation to resolve as a consequence of restored metabolic homeostasis [8]. This is achieved through multiple converging pathways: reductions in low-density lipoprotein (LDL) cholesterol, apolipoprotein B, and lipoprotein(a) that protect cerebrovascular integrity; increases in HDL particle number and functionality that enhance cholesterol efflux capacity and facilitate lipid transport from and between neurons and astrocytes; liberation of monomeric apolipoprotein A-I (ApoA-I) that might readily transport through the BBB from the plasma compartment and compensates for apoE4-HDL functional deficiencies; and elevated transport of lipophilic antioxidants from the plasma to the brain that protect against lipid peroxidation. This comprehensive lipid modulation addresses the root cause of APOE4-associated dysfunction, and the reductions in GFAP observed with obicetrapib in BROADWAY confirm that glial inflammation subsides once its metabolic trigger is removed.

The benefits of CETP inhibition in BROADWAY were not confined to APOE4 carriers. Among all participants, obicetrapib reduced p-tau217 progression by 2.84% relative to placebo (P=0.025), indicating that the intervention confers neuroprotective benefits relatively independent of APOE genotype [2]. While APOE4 carriers represent the highest-risk population with the greatest magnitude of benefit, the mechanistic pathways engaged by CETP inhibition, atherosclerosis prevention, enhanced HDL functionality, and antioxidant transport, address shared pathophysiology across the broader ASCVD population. Given that cardiovascular disease and AD share common vascular, metabolic, and inflammatory substrates, an intervention capable of addressing both disease processes through a single mechanism represents a compelling opportunity for patients facing concurrent risks [1].

The following review examines the biological foundation linking APOE status to disordered lipid metabolism in both peripheral and central compartments, the epidemiological and genetic evidence supporting CETP as a therapeutic target for dementia prevention, the mechanisms through which CETP inhibition may confer neuroprotection, and the clinical evidence establishing obicetrapib's effects on AD biomarkers. This synthesis supports the hypothesis that comprehensive lipid modulation through CETP inhibition represents a rational strategy for AD prevention; particularly, but not exclusively, for APOE4 carriers at highest genetic risk.

### 1.1 The interplay between apolipoprotein E status and peripheral and brain lipid metabolism in the pathogenesis of Alzheimer's disease

Disrupted lipid handling within the brain represents a fundamental driver of AD pathogenesis, manifesting through tightly linked disturbances in membrane composition, oxidative stress, and neuroinflammatory responses [9]. Neuronal and synaptic membranes depend heavily on cholesterol and phospholipids to regulate signal transduction, intracellular protein sorting, and immune surveillance within the CNS [10]. The consequences of aberrant lipid homeostasis between neurons and supporting glial populations are severe: abnormal neuronal firing patterns, deposition of Aβ into insoluble plaques, accumulation of hyperphosphorylated tau, and ultimately, irreversible neuronal death [11].

The biological foundation of this lipid imbalance traces to APOE status [12]. ApoE functions as the primary protein constituent of lipoprotein particles synthesized by astrocytes and microglia in the CNS. These CNS-derived particles exhibit remarkable structural similarity to peripheral high-density lipoproteins (HDL), containing phospholipids, unesterified cholesterol, and a cholesteryl ester (CE) core. Both peripheral HDL and brain-synthesized ApoE-HDL particles share core functions: removing excess cellular cholesterol and delivering lipophilic antioxidants to protect against oxidative damage [13].

These ApoE-HDL particles acquire their lipid cargo through interactions with cell surface ATP-binding cassette (ABC) transporters, particularly ABCA1 (facilitating free cholesterol efflux), ABCA7 (mediating phospholipid transport), and ABCG1, which are expressed on astrocytes, neurons, and microglia. Neuronal uptake of these apoE-coated lipoproteins occurs via receptor-mediated endocytosis through apoE binding to lipoprotein receptors, primarily the apoE receptor, the LDL receptor (LDLR), and low-density lipoprotein receptor-related protein 1 (LRP1) [14].

### 1.2 Apolipoprotein E genotypes and Alzheimer's disease risk

Three common allelic variants of the APOE gene: E2, E3, and E4, give rise to corresponding apoE protein isoforms that differ at two amino acid positions yet exhibit very different functional properties. The E3 allele predominates in most populations and serves as the reference against which other variants are compared. Carriers of E2 possess relative protection from AD, whereas E4 carriership constitutes the most powerful common genetic risk factor for AD. Heterozygous E4 carriers face roughly three-fold elevated risk; homozygotes face a striking 9- to 16-fold increase in lifetime AD probability [15].

APOE4 affects multiple neurodegenerative conditions beyond AD [16]. Proteomic studies show APOE4 carriers have similar immune changes across various neurodegenerative disorders. The APOE4 genotype demonstrates antagonistic pleiotropy. Evidence suggests that it may provide survival advantage in high-infection environments during youth, when acute pathogen exposure is elevated. While this enhanced immune response may protect younger E4 carriers from certain infections, this same hyperresponsive immune profile appears to become maladaptive with aging, as chronic low-grade inflammation becomes harmful. This is similar to what is observed in patients with ASCVD [17].

### 1.3 APOE4-mediated pathophysiology in Alzheimer's disease

The E4 allele's detrimental effects extend well beyond its inflammatory properties to include core defects in lipid shuttling and waste clearance. Astrocyte-to-neuron cholesterol delivery operates less efficiently in the presence of apoE4, and the protein's capacity to facilitate removal of oxidized lipid species from neurons and other CNS cell types is substantially compromised [18]. Neurons occupy a precarious position with respect to cholesterol: both deficiency and excess prove injurious, and this narrow homeostatic window explains why therapeutic strategies must enhance transport efficiency rather than simply reduce cholesterol levels [19]. When astrocytes fail to adequately export and process peroxidized lipids, these toxic species accumulate within neuronal compartments, triggering inflammatory cascades that accelerate cell death and drive progressive neurodegeneration [20]. These lipid transport defects do not occur in isolation. The brain's exceptionally high lipid content renders it vulnerable to oxidative modification, and the resulting oxysterols, unlike cholesterol itself, can cross the BBB bidirectionally [21]. This permeability establishes a self-reinforcing pathological loop: oxidative stress generates oxysterols such as 27-hydroxycholesterol, which flux into the brain from the peripheral circulation under hypercholesterolemic conditions; once present, these oxysterols promote further neuroinflammation, enhance Aβ generation, and trigger additional oxidative damage [22]. Glial activation compounds the insult, releasing reactive oxygen species and nitric oxide that perpetuate neuronal injury. ApoE4 appears to accelerate this cycle at multiple nodes, its impaired antioxidant function leaves neurons more susceptible to oxidative insult, while its deficient lipid efflux capacity allows toxic oxidized species to accumulate rather than be cleared. The net effect is a vicious circle in which dyslipidemia, oxidative stress, and neuroinflammation reinforce one another, driving disease progression in a manner that conventional LDL-lowering strategies may not adequately address. This self-perpetuating cascade has been characterized as the driving force behind AD development, with oxysterols serving as the critical link connecting peripheral hypercholesterolemia to central neurodegeneration; a connection that explains how systemic lipid disturbances can promote brain pathology despite the BBB's impermeability to cholesterol itself [6].

Work published recently has further implicated E4 homozygosity in pathological lipid droplet accumulation within microglia, adding another dimension to the metabolic dysfunction characteristic of this genotype [23].

The directionality of lipid transport failure has recently been clarifie d. While astrocyte-to-neuron cholesterol delivery has received considerable attention, neuron-to-astrocyte lipid efflux represents an equally critical pathway, and one profoundly impaired by apoE4. ApoE4 particles not only fail to extract oxidized unsaturated phospholipids from neurons but actively exacerbate their accumulation, triggering endolysosomal dysfunction and sensitizing neurons to ferroptosis. Particles containing apoE2 or the rare protective variant apoE3 Christchurch, by contrast, efficiently efflux these toxic lipid species through the ABCA7 transporter, rescuing endolysosomal function and restoring neuronal activity even in the presence of apoE4. Oxidized phospholipid clearance thus emerges as a genotype-dependent vulnerability, and therapeutic strategies that augment lipid efflux capacity, whether through enhanced apoA-I availability or other mechanisms, may prove particularly beneficial for E4 carriers [24].

The reach of apoE4-mediated dysfunction spans virtually every major CNS cell type: astrocytes, vascular endothelium, neurons, myelinating oligodendrocytes, and critically, microglia, whose functional parallels with arterial macrophages is well recognized. In E4 carriers, microglia adopt a distinctive phenotype marked by intracellular lipid loading and transcriptional upregulation of lipogenic pathways. The resulting metabolic dysfunction within these resident immune cells compromises their surveillance functions and disturbs the delicate balance of neuronal network activity, mirroring patterns observed in patients with AD [25].

Comprehensive lipidomic profiling of astrocytes derived from induced pluripotent stem cells has positioned cholesterol metabolism as a master regulator of glial immune function [22]. Astrocytes expressing apoE4 accumulate cholesterol esters to a far greater degree than their apoE3 counterparts, and this lipid burden directly shapes inflammatory capacity through interferon-mediated signaling networks. The downstream consequences include altered surface expression of major histocompatibility complex (MHC) class I antigens and modified immunoproteasome activity. Notably, when challenged with exogenous cholesterol or typical inflammatory triggers, TNF, IL-1α, or complement component C1q, apoE4-expressing astrocytes mount exaggerated immune responses relative to apoE3 controls. These observations reframe CE accumulation not as a passive byproduct of apoE4 dysfunction but as an active amplifier of neuroinflammatory signaling, offering a mechanistic rationale for how systemic lipid-modifying therapies might dampen inflammatory processes within the brain.

These combined defects in lipid synthesis and transport cause lipid accumulation within specific brain regions and enhanced Aβ aggregation. ApoE4-induced cholesterol dysregulation creates cell-specific effects that compromise neuronal synaptic function, alter glucose metabolism within astrocytes, promote microglial inflammatory responses, and impair oligodendrocyte remyelination, processes that substantially contribute to AD [22]. In the peripheral circulation, E4 carriers possess a distinctive lipid profile featuring elevated LDL-C, apolipoprotein B, and lipoprotein(a) levels, alongside reduced HDL cholesterol concentrations. These alterations significantly increase the risk of ASCVD while creating a metabolic environment that facilitates AD pathogenesis [26].

A recent genome-wide association study (GWAS) has provided novel insights into neurodegenerative disease pathogenesis. This investigation, stratified by apoE genotype, provided insights into previously unidentified loci implicated in multiple neurodegenerative conditions [27]. The identification of the DDHD1 gene, encoding a phospholipase A1 family member, further emphasizes the critical importance of lipid and phospholipid metabolism in AD [28].

### 1.4 High-density lipoproteins, cholesteryl-ester transfer protein, and Alzheimer's disease: epidemiological evidence

Epidemiological studies have shown consistent connections between HDL functionality, CETP activity levels, and long-term cognitive trajectories. Studies of exceptional longevity have identified genetic variants that reduce CETP function as correlates of preserved cognitive performance and diminished AD susceptibility, with particularly strong data in E4 carriers. Prospective cohort data demonstrate that carriers of the reduced-function V405 CETP polymorphism exhibit slower rates of memory decline and experience dementia at markedly lower rates, roughly 72% less frequently than non-carriers [29]. Complementary evidence links circulating apoA-I and HDL cholesterol concentrations to better cognitive test performance; meta-analysis of available data suggests that elevated apoA-I/HDL levels may reduce AD risk by approximately 15% in older adults [30].

CETP functions as a key regulator within systemic lipid networks by mediating the movement of CE between HDL and apoB-containing lipoproteins [21,22]. Through this transfer mechanism, CETP reduces HDL cholesterol concentrations while elevating LDL-C levels, creating a more pro-atherogenic lipid environment. Although CETP appears minimally expressed in cerebral tissue, primarily within astrocytes, transgenic mice studies reveal that enhanced systemic CETP function markedly influences brain cholesterol homeostasis. These investigations show that CETP-overexpressing mice develop approximately 22% greater cerebral cholesterol accumulation, following dietary cholesterol exposure, underlining CETP's significant peripheral influence on CNS lipid regulation [31].

Clinical observations from Japan, though limited in scope, have documented a strikingly low incidence of ASCVD, AD, and age-related macular degeneration among individuals homozygous for loss-of-function CETP mutations, a natural experiment suggesting broad protective effects of lifelong CETP absence [32].

Mendelian randomization approaches have further strengthened the case for a causal relationship between CETP activity and dementia. An analysis of more than one million participants across five independent cohorts found that genetically determined reductions in CETP function associated with meaningfully lower all-cause dementia risk. Importantly, this protective signal persisted regardless of whether individuals carried zero, one, or two E4 alleles, demonstrating that CETP-mediated risk modification operates through pathways at least partially distinct from apoE. The implication is that sustained pharmacological CETP inhibition might reproduce these protective genetic effects, supporting the biological rationale for prevention-focused clinical development [33].

These genetic findings warrant consideration of phenotype-specific effects. MR analyses of over one million participants demonstrated that reduced CETP function was associated with lower risk across dementia subtypes, including Alzheimer's disease (OR 0.68), vascular dementia (OR 0.30), and unspecified dementia (OR 0.15) [33]. Separate analyses using autopsy-confirmed cases found protective effects for Lewy body dementia (OR 0.81) and Parkinson's disease dementia (OR 0.26), with effects most pronounced in APOE4 carriers [34]. The convergent findings across multiple dementia phenotypes support the biological plausibility of CETP inhibition for neurodegeneration. The majority of available genetic data derive from European-ancestry populations; whether these associations replicate in diverse ancestry groups remains to be established.

CETP inhibition provides a novel therapeutic strategy for AD prevention that differs from conventional approaches. Unlike interventions targeting the downstream consequences of pathological processes such as amyloid plaque removal, CETP inhibition comprehensively modulates foundational lipid imbalances, oxidative damage, and inflammatory processes that may initiate disease development, with relevance for APOE4 carriers. This therapeutic mechanism appears to function through multiple integrated biological pathways that collectively address APOE4-associated neurodegeneration [34].

### 1.5 CETP inhibition as a therapeutic strategy for Alzheimer's disease prevention

How can reduction of peripheral CETP activity translate into multiple effects that counteract apoE4 allele presence?

### 1.6 Previous cholesterol-lowering approaches in Alzheimer's disease

Earlier investigations of cholesterol-lowering medications in patients with AD yielded disappointing outcomes. Simvastatin was tested in the CLASP-AD trial, and atorvastatin in the LEADe study, both involving patients with mild to moderate AD, yet neither demonstrated cognitive benefits [35,36]. Several fundamental differences distinguish CETP inhibition from these earlier statin interventions.

Statins reduce cholesterol primarily by blocking hepatic synthesis, which lowers LDL-C and provides modest reductions in other atherogenic lipoproteins but does not meaningfully raise HDL-C levels. In contrast, CETP inhibition operates through distinct mechanisms that extend beyond LDL-C reduction. It not only reduces LDL-C and other atherogenic particles but substantially increases functional HDL-C while improving HDL particle quality [52,53,59]. These effects may confer neuroprotection through antioxidant and anti-inflammatory mechanisms. Importantly, CETP is expressed, to some extent, in brain tissue itself, particularly in astrocytes where it directly modulates CNS cholesterol balance, a pathway that is distinct from the peripheral LDL reduction statins achieve. Genetic studies lend support to this mechanistic difference: populations with lower CETP activity have demonstrated better cognitive preservation and reduced dementia risk, whereas similar protective associations have not been established for statin-targeted pathways [37].

### 1.7 Mechanisms of neuroprotection through CETP inhibition

The cardiovascular benefits of CETP inhibition translate directly to cerebrovascular protection through anti-atherosclerotic mechanisms. Through LDL-C reduction, HDL-C elevation, and lipoprotein(a) lowering, CETP inhibition establishes a lipid environment that might support BBB integrity and facilitates improved molecular transport across this interface. Improved lipids and lipoproteins support cerebrovascular health by preventing arterial atherosclerosis, maintaining optimal brain perfusion, and potentially reducing both vascular cognitive impairment and AD-associated pathology [38].

### 1.8 Blood-Brain Barrier Integrity

The BBB represents a critical interface where peripheral lipid metabolism directly influences CNS health. In APOE4 carriers, this barrier shows early and progressive dysfunction that appears independent of classical AD pathology, positioning vascular compromise as a potential initiating event rather than a downstream consequence of neurodegeneration.

Neuroimaging studies have demonstrated that cognitively normal APOE4 carriers exhibit increased BBB permeability in the hippocampus and parahippocampal gyrus compared to non-carriers, even in the absence of detectable amyloid or tau pathology [39]. Elevated cerebrospinal fluid markers of pericyte injury predict subsequent cognitive decline specifically in APOE4 carriers, suggesting that microvascular damage precedes and potentially partly drives neuronal dysfunction in this population.

The cellular basis for this vulnerability traces to astrocyte-derived apoE4. Experimental models demonstrate that astrocytic expression of the E4 isoform actively disrupts barrier integrity through tight junction degradation, reduced astrocyte end-foot coverage of cerebral microvessels, and elevated matrix metalloproteinase-9 (MMP9) activity [40]. Importantly, this represents a gain-of-toxic-function: removing astrocytic apoE4 rescues barrier integrity within weeks, whereas apoE3 deletion produces no such effect. This finding indicates that apoE4 does not simply fail to support BBB maintenance but actively promotes its breakdown.

The mechanistic pathway also centers on altered interactions between apoE4 and LRP1 [40]. Under normal conditions, apoE binding to LRP1 on pericytes suppresses cyclophilin A signaling and downstream MMP9 activation. ApoE4′s reduced LRP1 binding affinity releases this inhibitory control, permitting cyclophilin A to activate nuclear factor-κB and drive MMP9 expression. The resulting proteolytic cascade degrades tight junction proteins and basement membrane components. Plasma proteins including fibrinogen and thrombin then extravasate into brain parenchyma, where they activate additional inflammatory pathways that perpetuate barrier dysfunction through secondary MMP9 release.

Clinical observations confirm a dose-dependent relationship between APOE4 allele number and BBB permeability across neurodegenerative conditions [41]. Homozygous E4/E4 carriers demonstrate the greatest barrier compromise, heterozygotes show intermediate effects, and non-carriers maintain relative barrier integrity. These differences persist after adjustment for age, disease severity, and vascular comorbidities, indicating a genotype-specific effect on cerebrovascular function.

The functional consequence of impaired BBB integrity extends to Aβ clearance mechanisms. Endothelial cells normally transport Aβ from brain to blood through LRP1 and p-glycoprotein-dependent pathways, but APOE4 expression substantially reduces this transport capacity [42]. MMP9 activity further exacerbates clearance deficits by shedding endothelial LRP1, creating a self-perpetuating cycle: barrier dysfunction impairs Aβ removal, accumulated Aβ promotes further inflammation and MMP activation, and the resulting proteolytic environment degrades additional clearance machinery. Notably, APOE2 demonstrates protective effects on these same transport systems, consistent with its association with reduced AD risk.

These findings establish BBB dysfunction as an early event in APOE4-associated neurodegeneration, occurring upstream of classical amyloid and tau pathology. This temporal primacy suggests that interventions targeting cerebrovascular health may offer a critical window for disease modification before irreversible neuronal loss occurs. CETP inhibition may address this vulnerability through multiple converging mechanisms: HDL particles exert direct protective effects on endothelial cells by accepting and inactivating oxidized phospholipids, reducing reactive oxygen species generation through NADPH oxidase inhibition, suppressing inflammatory signaling, and maintaining tight junction integrity. Additionally, CETP inhibition increases HDL-associated sphingosine-1-phosphate (S1P), which activates endothelial S1PR1 receptors to reinforce barrier stability and counteract the pro-permeability effects of the CypA-MMP9 cascade [40]. The molecular basis for this protection has been further characterized. Apolipoprotein M (ApoM), which anchors to HDL particles via its unprocessed signal peptide, serves as the primary carrier of plasma S1P, with approximately 60–70% of circulating S1P bound to ApoM-containing HDL. When delivered by ApoM rather than albumin, S1P preferentially activates the S1PR1-Rac1 pathway that stabilizes tight junctions while avoiding engagement of S1PR2/3-RhoA signaling that promotes endothelial contraction and barrier disruption; ApoM-deficient mice demonstrate significantly increased BBB permeability, confirming the critical role of HDL-associated S1P transport in maintaining barrier integrity [63]. By enhancing HDL particle number and functionality, CETP inhibition may counteract the pro-inflammatory, pro-proteolytic environment that apoE4 creates at the neurovascular level, potentially interrupting the pathological cascade before it propagates to neuronal compartments.

### 1.9 HDL/apoA-I

Central to the hypothesis that CETP inhibition affects brain health is its impact on HDL particles. CETP inhibition generates substantial HDL cholesterol elevations concurrent with HDL particle enlargement. During this growth process, HDL particles incorporate circulating apoE while simultaneously releasing monomeric, lipid-free apoA-I. The acquired apoE functions as a ligand for multiple receptors including scavenger receptor B1 (SR-B1), LDLR, and LRP1, facilitating enhanced hepatic HDL processing. While apoE-enriched HDL undergoes enhanced hepatic uptake via these receptors, CETP inhibition produces net HDL-C elevation by preventing CE transfer to ApoB lipoproteins, with cholesterol remaining in the HDL pool. Concurrently, liberated lipid-free apoA-I triggers the HDL cascade via monomeric apoA-I, pre-beta1, and pre-beta2 HDL formation [43].

Recent work has established that lipid-free apoA-I enters cerebrospinal fluid with relative ease, [44]. raising the possibility that these early-stage HDL precursors might offset the functional shortcomings of apoE4-containing particles with respect to cholesterol efflux, phospholipid mobilization, Aβ clearance, and neuroinflammatory control. Concurrent investigations have identified peripherally derived apoA-I as a circulating signal capable of directly influencing microglial behavior through regionally selective uptake. Tracer studies employing fluorescent-tagged plasma proteins revealed that microglial subpopulations concentrated in the hypothalamus, thalamus, and hippocampus preferentially capture blood-borne apoA-I via SR-B1. Microglia that have internalized plasma-derived factors display higher metabolic activity, better phagocytic function, and elevated transcription of genes governing innate immune function and antigen processing relative to their plasma-negative neighbors [45].

Animal studies utilizing peripheral overexpression of human apoA-I have demonstrated preservation of cognitive performance, reduction in neuroinflammatory markers, and protection against cerebral amyloid angiopathy, findings that implicate circulating small HDL particles as direct participants in the clearance of Aβ from the CNS vasculature and parenchyma [46].

Functional studies have confirmed that microglial internalization of apoA-I improves their ability to engulf Aβ aggregates and myelin debris while simultaneously dampening inflammatory activation under conditions of cellular stress [47]. Mice lacking apoA-I exhibit pronounced deficits in hypothalamic microglial phagocytosis, impairments that resolve following systemic apoA-I repletion. Separate lines of evidence show that apoE4 expression drives cholesterol dysregulation and lipid accumulation within glia, whereas pharmacological or genetic upregulation of ABCA1 confers substantial neuroprotection and reduces tau burden. Because apoA-I binds ABCA1 with higher affinity than apoE4-containing particles, increasing CNS apoA-I availability may prove especially beneficial for E4 carriers by restoring efficient cholesterol efflux and limiting inflammatory activation. ABCA7 has also been implicated as a critical transporter for oxidized phospholipid efflux from neurons, a pathway through which protective ApoE variants appear to exert their beneficial effects [24]. The functional parallels between microglia and peripheral tissue macrophages suggest that apoA-I exerts these beneficial effects through conserved pathways governing cholesterol and phospholipid handling, establishing a direct link between improvements in systemic HDL function and neuroprotection in genetically susceptible individuals [48].

The small, functionally active HDL particles generated through CETP inhibition engage several neuroprotective mechanisms with particular relevance to CNS cholesterol balance. ApoA-I, the principal functional constituent of HDL, facilitates Aβ removal both through direct binding interactions and by enhancing clearance pathways,[49]. while apoE-containing HDL-like particles coordinate broader aspects of brain cholesterol trafficking [50]. For individuals carrying the E4 allele, the improved HDL functionality achieved through CETP inhibition may help restore cholesterol homeostasis and counteract genotype-associated risk. Importantly, these smaller HDL species, whose concentrations rise substantially with CETP inhibition, can cross the BBB endothelium via intact particle uptake mechanisms and exert local anti-inflammatory effects [51].

### 1.10 Lipophilic Antioxidants

A further dimension of CETP inhibition involves its effects on lipophilic antioxidant transport. Concentrations of lutein, zeaxanthin, and α-tocopherol rise in both circulating HDL fractions and cerebrospinal fluid following CETP inhibition, reflecting enhanced delivery for these protective molecules into central tissues [52,53]. These carotenoids and vitamin E species serve as scavengers of reactive oxygen species and inhibitors of lipid peroxidation, processes that otherwise fuel neuroinflammatory cascades. Lutein can also bind directly to Aβ peptides, potentially interfering with their aggregation into neurotoxic assemblies [54]. Clinical correlation studies have linked higher serum carotenoid levels to better cognitive test performance and reduced dementia severity, with affected individuals demonstrating 20–30% improvements on standardized assessments alongside lower concentrations of neuroinflammatory markers [55].

**Fig. 1:**
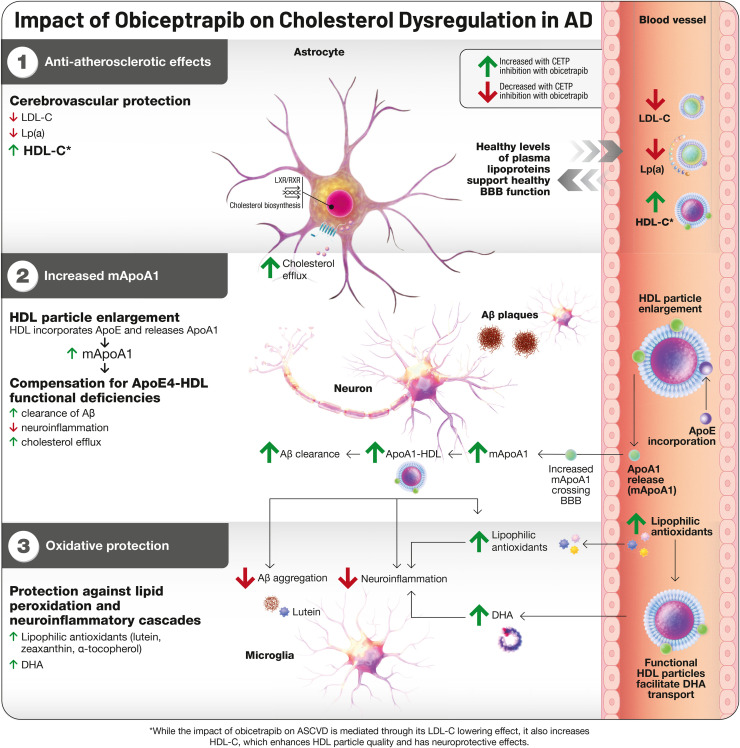
CETP inhibition: A Potential Pathway for Targeting Cholesterol Dysregulation in AD. ABCA, ATP-binding cassette subfamily A; Aβ peptide, amyloid beta peptide; AD, Alzheimer's disease; Apo, apolipoprotein; CETP, cholesteryl ester transfer protein; HC, hydroxycholesterol; HDL, high-density lipoprotein; LCAT, lecithin-cholesterol acyltransferase; LDL, low-density lipoprotein; LDLR: low-density lipoprotein receptor; LRP1, LDL-receptor-related protein 1; PLTP, phospholipid transfer protein; LA, lipophilic antioxidants 1. The cardiovascular benefits of obicetrapib's lipid modulation effects translate directly to cerebrovascular protection through anti-atherosclerotic mechanisms. 2. In addition, obicetrapib increases monomeric apoA-I levels, which can cross the blood-brain barrier and enter cerebrospinal fluid. This becomes relevant for APOE4 carriers, whose lipid transport systems work less efficiently. The increased brain apoA-I helps compensate by improving cholesterol and phospholipid removal from neurons, assisting with Aβ clearance, and reducing neuroinflammation, functions that APOE4 performs inadequately. 3. Concurrently, obicetrapib increases concentrations of lipophilic antioxidants, including lutein, zeaxanthin, and alpha tocopherol, in both peripheral HDL particles and brain fluid. These antioxidants protect against lipid peroxidation and reactive oxygen species that trigger inflammatory cascades in neurodegeneration and function as anti-aggregation modalities. This approach, enhancing lipid transport while delivering antioxidant protection, addresses both the transport deficiencies and oxidative stress that contribute to APOE4-associated brain pathology. These converging pathways suggest that CETP inhibition targets upstream metabolic problems rather than late downstream disease symptoms, potentially offering protection for individuals genetically predisposed to lipid-mediated neurodegeneration. Note: Pathways depicted represent a synthesis of established mechanisms (e.g., CETP effects on lipid levels, apoA-I release during HDL remodeling) and hypothesized neuroprotective effects (e.g., direct CNS benefits of increased apoA-I availability) that require further validation in humans.

Last, HDL facilitates omega-3 fatty acid transport, particularly docosahexaenoic acid (DHA), to cerebral tissues, where HDL-associated carotenoids prevent polyunsaturated fatty acid (PUFA) oxidation. Through small functional HDL particle enhancement and brain carotenoid elevation, CETP inhibition may protect cerebral PUFAs including DHA [56]. This antioxidant transport function assumes particular importance given that oxysterols generated through lipid peroxidation can cross the BBB and directly promote Aβ accumulation, tau hyperphosphorylation, and glial activation, processes that lipophilic antioxidants may help interrupt at their source. (Fig. 1).

### 1.11 Obicetrapib: Clinical development and Alzheimer's disease biomarker effects

Obicetrapib inhibits CETP activity by approximately 98%, effectively creating conditions resembling homozygous CETP deficiency [57]. The clinical development program for obicetrapib has included extensive phase 3 trials involving over 3,200 patients, establishing both its lipid-modulating efficacy and favorable safety profile.

In a preliminary proof-of-concept (POC) study, 13 APOE4-positive patients with mild cognitive impairment and elevated AD biomarkers were treated with obicetrapib, resulting in reduced plasma and cerebrospinal fluid levels of 24S- and 27-hydroxycholesterol (HC), increased concentrations of essential lipophilic antioxidants and carotenoids, and stabilization of key biomarkers indicative of AD pathology (NCT05161715) [58]. Evidence from previous phase 2 studies has shown that obicetrapib treatment resulted in significant increases in plasma concentrations of apoE, apoA-I, monomeric apoA-I, pre-beta1 particles, and pre-beta2 particles [52,53,59]. Obicetrapib also increased lipophilic antioxidant concentrations in both plasma HDL fractions and cerebrospinal fluid [52,53]. The observed reduction in 24-HC levels warrants particular attention. Cytochrome P450 46A1 (CYP46A1) shows high expression in pyramidal neurons of the cortex and hippocampus, and this enzyme accounts for 98-99% of cerebral 24-HC production. As the major physiological route for eliminating excess cholesterol from the brain, CYP46A1 generates 24HC that readily diffuses across the BBB into systemic circulation for final biliary elimination [60]. Given this pathway's dominant role, the decrease in 24HC levels following obicetrapib treatment points to engagement in brain cholesterol homeostasis.

The pathways through which obicetrapib produces its effects on AD biomarkers have not been fully characterized. The proof-of-concept study demonstrated reductions in CSF 24-hydroxycholesterol, suggesting engagement with brain cholesterol metabolism. However, whether this reflects direct CNS penetration or indirect effects mediated through peripheral lipid modulation, improved cerebrovascular health, or enhanced transport of neuroprotective molecules across the BBB remains to be determined. The strong correlation between obicetrapib plasma concentrations and p-tau217 changes (r=−0.64) supports CETP inhibition as a mechanism, but future studies directly measuring CETP activity and lipoprotein particle characteristics will help clarify the relative contribution of central versus peripheral pathways.

The convergence of biological, genetic, and Mendelian randomization data, together with the recognition that ASCVD patients represent an accessible high-risk population already under medical surveillance, provided the scientific rationale for incorporating a prespecified AD biomarker analysis into the obicetrapib BROADWAY phase 3 trial (ClinicalTrials.gov: NCT05142722) [61].

The BROADWAY trial provided clinical evidence supporting obicetrapib's efficacy in high-risk cardiovascular patients through a randomized, placebo-controlled study that enrolled 2530 participants with ASCVD or HeFH already receiving maximum tolerated lipid-lowering therapy. Treatment with obicetrapib produced significant improvements in multiple lipid parameters compared to placebo, while safety analyses demonstrated comparable adverse event profiles between treatment groups. The study showed no concerning patterns for diabetes, liver dysfunction, or muscle-related adverse effects, and investigators observed favorable trends for new-onset diabetes mellitus and cardiovascular outcomes. This evidence positions obicetrapib as a promising addition to existing cardiovascular risk management strategies [61,62].

The prespecified analysis revealed that obicetrapib significantly modified AD biomarker trajectories over 12 months, with treatment effects observed across tau (p-tau217, p-tau181), amyloid (Aβ42:40), neurodegeneration (NfL), and neuroinflammation (GFAP) markers. Effects were most pronounced in E4/E4 homozygotes (Table 2). Notably, higher baseline p-tau217 concentrations predicted faster progression in placebo recipients but corresponded with greater reductions in the obicetrapib group (P interaction<0.0001), suggesting the intervention provides maximal benefit to those with active pathology. Participants in the obicetrapib arm also had a significant decrease in conversion to elevated p-tau217 defined by the threshold of 0.42 pg/mL (P=0.004) (Table 2) [2].

**Table 2:** Percent change in AD biomarkers among E4/E4 participants.

Biomarker	Obicetrapib (%)	Placebo (%)	Difference (%)	P-value
p-tau217	−7.81	+12.67	−20.48	0.010
NfL	−10.49	+6.82	−17.31	0.020
GFAP	−6.39	+8.85	−15.24	0.006
p-tau181	−10.51	+3.16	−13.67	0.06
Aβ42:40	−0.36	−8.32	+7.96	0.013
p-tau217/Aβ42:40	−1.67	+20.98	−22.65	0.032

Values represent mean percent change from baseline. Negative values for p-tau217, NfL, GFAP, and p-tau181 indicate reduced biomarker progression with obicetrapib. Positive value for Aβ42:40 indicates favorable effect (higher ratio reflects reduced amyloid burden). NfL, neurofilament light chain; GFAP, glial fibrillary acidic protein; Aβ42:40, amyloid-beta 42:40 ratio.

### 1.12 Limitations of the BROADWAY biomarker analysis

Several limitations of the BROADWAY biomarker data warrant consideration. First, although ApoE phenotyping was unknown for 35% of participants, baseline characteristics were generally similar between the analysis population and those excluded, with differences primarily limited to lipids/lipoproteins, suggesting that excluded participants did not compromise external validity. Second, the small number of ApoE4 homozygotes (n=29) limits effect estimate precision in this subgroup despite observed statistical significance. Third, cognitive function and clinical outcomes were not assessed; the clinical significance of these biomarker changes can only be established through prospective trials that include cognitive testing. Fourth, the 12-month treatment duration, while adequate for detecting biomarker changes, may not fully capture long-term biomarker trajectory. Fifth, plasma AD biomarkers including p-tau217 have strong validation against CSF and PET measures, but their performance characteristics specifically in ASCVD populations require further study. Finally, these findings require confirmation in a dedicated trial designed to test the AD prevention hypothesis with cognitive endpoints.

However, important limitations apply to biomarker-based approaches. Plasma p-tau217 and Aβ42:40 ratio have demonstrated strong correlations with PET-confirmed pathology and predict future cognitive decline, but they have not been formally validated as surrogate endpoints for regulatory approval in AD prevention trials. The relationship between biomarker modification and clinically meaningful cognitive preservation remains to be established through long-term studies. Ongoing trials, including TRAILBLAZER-ALZ 3 evaluating donanemab in cognitively unimpaired individuals with amyloid and tau pathology, will provide important insights into the utility of plasma biomarkers in guiding AD prevention drug development [64]. Regulatory agencies including FDA and EMA have indicated openness to biomarker-enriched trial designs for accelerated development pathways, but demonstration of clinical benefit remains necessary for full approval [65].

### 1.13 Implications for preventive cardiology practice

For preventive cardiologists, these findings raise important practical considerations. Emerging data demonstrate that cognitive impairment and AD pathology are substantially under-recognized in cardiovascular populations. A recent report demonstrated that 29% of patients with ASCVD had cognitive performance consistent with MCI on standardized testing despite lacking formal diagnoses, and 55% had elevated p-tau217 levels indicative of AD pathology [3]. This prevalence substantially exceeds general population estimates; a recent large population-based study using plasma p-tau217 found that only 23.5% of cognitively unimpaired individuals aged 70 and older had evidence of AD neuropathological changes [66]. The nearly two-fold higher prevalence of elevated p-tau217 in ASCVD populations compared to the general population underscores the disproportionate burden of preclinical AD pathology in patients already engaged in cardiovascular care.

Furthermore, the population-based data confirm the strong influence of APOE genotype, with AD pathology prevalence increasing from 27.1% in non-carriers to 46.4% with one E4 allele and 64.6% with two E4 alleles [66]. This gradient reinforces the rationale for APOE4-enriched prevention strategies.

Patients with ASCVD thus represent an accessible population already engaged in longitudinal care, many of whom carry unrecognized cognitive risk. While routine AD biomarker screening cannot yet be recommended outside of research settings, clinicians should recognize that aggressive lipid management may confer benefits extending beyond cardiovascular protection. The emergence of validated plasma biomarkers such as p-tau217 may eventually enable risk stratification in cardiovascular clinics. In the interim, optimizing lipid profiles in APOE4 carriers, who demonstrate both elevated cardiovascular and cognitive risk, represents a reasonable approach consistent with current evidence, though dedicated trials will be required before specific recommendations regarding CETP inhibition for cognitive protection can be made.

## 2 Future directions

Several key questions should guide future research. The magnitude of biomarker effects observed in BROADWAY was greatest among APOE4 carriers, suggesting this population, particularly those with preclinical AD defined by elevated p-tau217 but without cognitive impairment, represents a logical target for dedicated prevention trials. Per FDA guidance on Early Alzheimer's Disease drug development and NIA-AA criteria, such individuals may demonstrate treatment effects on biomarkers while retaining sufficient cognitive reserve to show meaningful clinical benefit [65].

Biomarker screening should precede enrollment to enrich for individuals with active preclinical pathology who are most likely to benefit from intervention. Cognitive endpoints using validated composites such as the Preclinical Alzheimer's Cognitive Composite with Semantic Fluency (PACC5) are feasible in preclinical populations, though extended follow-up may be required to demonstrate meaningful effects. Biomarker-based endpoints can demonstrate biological activity more rapidly and may support accelerated development pathways. If ongoing secondary prevention trials such as TRAILBLAZER-ALZ 3 demonstrate that amyloid and tau biomarker effects are associated with clinical benefit, regulatory agencies could consider these measures as surrogate endpoints reasonably likely to predict clinical benefit in other prevention trials [67]. The convergence of cardiovascular and cognitive risk in ASCVD populations creates an opportunity for pragmatic prevention trials that address both disease processes.

## 3 Synthesis and path forward

The findings reviewed here support a coherent biological framework linking CETP activity to AD pathophysiology. Genetic and epidemiological evidence consistently associates reduced CETP function with preserved cognition and lower dementia risk, while mechanistic studies have identified the pathways through which CETP inhibition could address lipid trafficking deficits characteristic of APOE4-associated neurodegeneration: enhanced cholesterol efflux capacity, improved apoA-I availability in the CNS, augmented antioxidant transport, and protection of BBB integrity. The BROADWAY biomarker analysis provides prospective, randomized, clinical evidence that pharmacological CETP inhibition with obicetrapib modifies AD biomarker trajectories across tau, amyloid, neurodegeneration, and neuroinflammatory markers, with the greatest magnitude of benefit observed in those at highest genetic risk. These data demonstrate engagement with biological pathways central to disease pathogenesis. Notably, CETP inhibition with obicetrapib may interrupt the pathological cycle whereby peripheral hypercholesterolemia generates oxysterols that cross the BBB and promote neuroinflammation, Aβ accumulation, and oxidative damage, a self-amplifying cascade proposed as a central driver of AD development and one particularly accelerated in APOE4 carriers.

Although BROADWAY enrolled patients with cardiovascular disease, the mechanistic rationale for CETP inhibition in AD prevention extends to any individual at elevated risk, whether defined by APOE genotype, age, family history, or emerging biomarker profiles. The shared lipid biology linking cardiovascular and neurodegenerative disease suggests broad applicability rather than restriction to a cardiovascular population. Individuals without cardiovascular disease but with genetic susceptibility or early biomarker elevations represent an equally relevant target for preventive intervention.

**Fig. 2:**
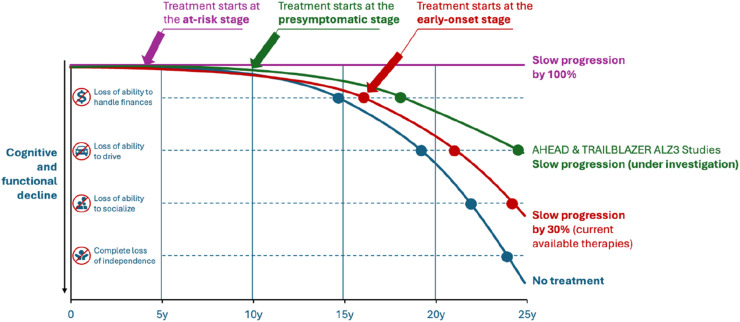
Treatment of high-risk patients before clinical evidence of ad could prevent decline and halt disease progression 1. The natural history of AD involves progressive functional loss over approximately 25 years (blue curve). 2. Current anti-amyloid therapies initiated at the early symptomatic stage slow progression by approximately 30% (red curve). 3. Trials evaluating presymptomatic intervention (AHEAD, TRAILBLAZER-ALZ 3) aim to demonstrate greater benefit in biomarker-positive but cognitively normal individuals (green curve). 4. Intervention at the at-risk stage, before substantial pathology accumulates, offers the theoretical potential to halt progression entirely (purple curve). 5. This framework underscores why biomarker-based endpoints may be necessary for early-stage trials: cognitive decline cannot be measured in individuals who are not yet declining.

Emerging consensus in the field recognizes that timing of intervention fundamentally determines both what can be achieved and how benefit should be measured. AD pathology accumulates over 15 to 20 years before the onset of cognitive symptoms. Interventions initiated at the early symptomatic stage have demonstrated modest effects, slowing progression by approximately 30% in recent trials of anti-amyloid therapies. Trials such as AHEAD and TRAILBLAZER-ALZ 3 are now evaluating intervention at the presymptomatic stage, in individuals with biomarker evidence of pathology but intact cognition, with the expectation of greater benefit. Extending this logic further, intervention at the at-risk stage, before substantial pathology has accumulated, offers the greatest potential for altering disease course, potentially halting progression rather than merely slowing decline already underway (Fig. 2).

This trajectory has implications for endpoint selection. Cognitive decline cannot be measured in individuals who are not declining. For populations at the earliest disease stages, cognitively normal individuals identified through genetic risk or subtle biomarker elevations, traditional cognitive endpoints may lack sensitivity within practical trial durations. Biomarker-based endpoints can demonstrate biological effects in precisely these populations, where the goal is prevention rather than treatment of established disease. The BROADWAY observation that higher baseline p-tau217 concentrations predicted greater treatment benefit suggests that identifying individuals with active but early pathology may define an optimal intervention window, early enough that neuronal loss has not yet occurred, but with sufficient biological activity to detect and modify.

Practical considerations also favor evaluation of oral, well-tolerated interventions in the context of population-level prevention. Current disease-modifying approaches under investigation require parenteral administration, specialized infusion centers, and monitoring for amyloid-related imaging abnormalities. These requirements present barriers to deployment as preventive therapy across large populations. An oral agent with an established safety profile could enable earlier and broader intervention, reaching individuals in primary care settings where cardiovascular risk is already being managed, before they present to specialized memory clinics.

## 4 Conclusion

Genetic evidence, biological plausibility, and clinical biomarker data have now converged to suggest CETP inhibition as a candidate for further evaluation in AD prevention. Critical questions remain regarding optimal target populations, timing of intervention, and endpoints capable of capturing meaningful biological effects in individuals who remain cognitively intact. Addressing these questions will require trials designed for prevention rather than treatment, but the rationale for conducting such trials rests on substantially stronger ground than it did prior to BROADWAY.

**Central Illustration**

**Figure:**
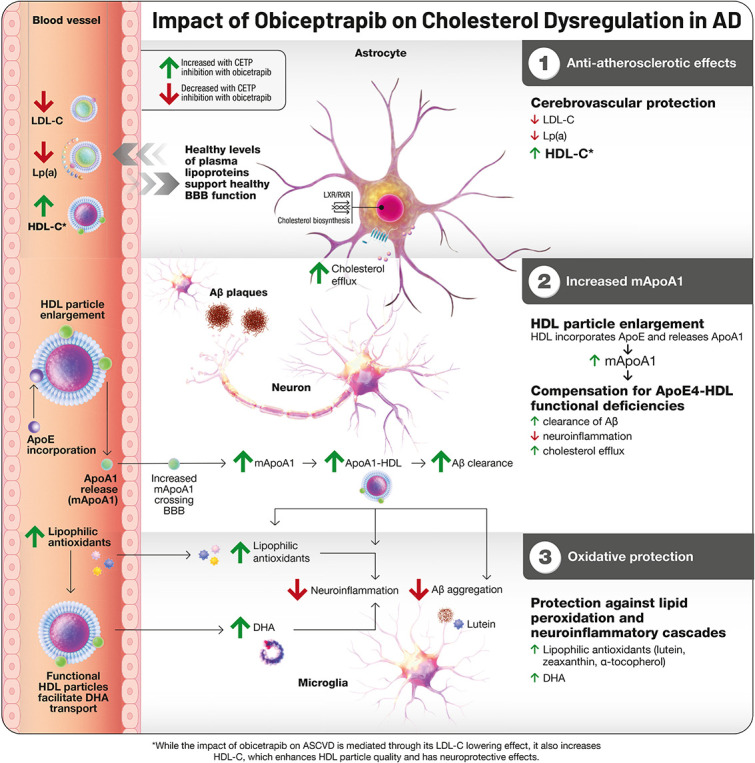

Data Availability and Independent Analysis Statement

The BROADWAY biomarker analyses were conducted using data from the BROADWAY trial (NCT05142722). Biomarker assays were performed by a qualified central laboratory (Medpace Reference Lab) using validated commercial assays. Statistical analyses were conducted independently by an academic statistician (M.S.) who had access to the complete dataset. The authors affiliated with NewAmsterdam Pharma had access to study data; interpretation of results was developed collaboratively with academic co-authors who provided independent scientific oversight.

## CRediT authorship contribution statement

**Michael H Davidson:** Writing – review & editing, Writing – original draft. **Andrew Hsieh:** Writing – review & editing, Writing – original draft. **Mathijs de Kleer:** Writing – review & editing, Writing – original draft. **Michael S. Szarek:** Writing – review & editing, Writing – original draft, Data curation. **Philip Scheltens:** Writing – review & editing, Writing – original draft. **Everard Vijverberg:** Writing – review & editing, Writing – original draft. **Adam Johnson:** Writing – review & editing, Writing – original draft. **Marc Ditmarsch:** Writing – review & editing, Writing – original draft. **John J.P. Kastelein:** Writing – review & editing, Writing – original draft.

## Declaration of competing interest

Michael Szarek: reports receiving salary support from CPC, a nonprofit academic research organization affiliated with the University of Colorado, that receives or has received research grant/consulting funding between July 2021 and July 2024 from the following organizations: Abbott Laboratories, Agios Pharmaceuticals, Inc., Alexion Pharma Godo Kaisha, Amgen Inc., Anthos Therapeutics, Inc., ARCA biopharma, Inc., Arrowhead Pharmaceuticals, AstraZeneca Pharma India, AstraZeneca UK Ltd, Bayer, Bayer Aktiengesellschaft, Bayer Pharma AG, Beth Israel Deaconess Medical Center, Better Therapeutics, Boston Clinical Research Institute, LLC, Bristol-Myers Squibb, Cleerly, Inc., Colorado Dept of Public Health and Environment, Congress Inc, Cook Regentec LLC, CSL Behring LLC, Eidos Therapeutics, Inc., EPG Communication Holdings Ltd., Esperion Therapeutics, Inc, Faraday Pharmaceuticals, Inc., HeartFlow Inc, Insmed, Ionis Pharmaceuticals, IQVIA Inc., Janssen Pharmaceuticals, Inc, Janssen Research & Development, LLC, Janssen Scientific Affairs LLC, Lexicon Pharmaceuticals, Inc., Medpace, Inc., Medscape, Merck Sharp & Dohme Corp., Nectero Medical, Inc, Novartis Pharmaceuticals Corporation, Novo Nordisk Inc., Pfizer, PPD Development, L.P., Prothena Biosciences Limited, Regeneron, Regents of the University of Colorado (aka UCD), Sanifit Therapeutics S.A., Sanofi, Silence Therapeutics PLC, Stanford University, Stealth BioTherapeutics Inc., The Brigham and Women’s Hospital, Thrombosis Research Institute, Tourmaline Bio, Inc, University of Colorado, University of Colorado Denver, University of Pittsburgh, VarmX, Verve Therapeutics, WraSer, LLC. Dr. Szarek also reports serving as a consultant or research support (or both) from Amarin, Lexicon, NewAmsterdam, Novartis, Regeneron, Sanofi, Silence, and Tourmaline.

Everard Vijverberg: received consultancy fees (paid to the university) for New Amsterdam Pharma, Treeway, ReMynd, Vivoryon, Biogen, Vigil Neuroscience, ImmunoBrain Checkpoint, Muna Therapeutics, Esai, Eli Lilly, CogRX, Therini, UCB and Roche. Within his university affiliation he is PI of studies of DIAN, AC immune, Alnylam, CogRX therapeutics, New Amsterdam Pharma, Janssen, UCB, Roche, Vivoryon, ImmunoBrain, GSK, MSD, Biogen, Alector, Eli Lilly, AriBio Fuij Film Toyama, GemVax. Co-founder van het CANDIDATE Center Amsterdam UMC. Scientific projects with the Dutch Soccer Association (KNVB)

Philip Scheltens: is a full time employee of EQT LifeSciences, emeritus prof at Amsterdam University Medical Center, and consultant to New Amsterdam Pharma

Michael H Davidson, John JP Kastelein, Mathijs de Kleer, Marc Ditmarsch, and Andrew Hsieh are employees of NewAmsterdam Pharma and hold stocks or options.
